# Supplementary material for: Novel start codons introduce novel coding sequences in the human genomes
Source: Sci Rep. 2023 May 19;13:8141. doi: 10.1038/s41598-023-34770-z (PMC10198996; doi:10.1038/s41598-023-34770-z)
Supplement: Supplementary file 1 — Supplementary Information 1. [file 41598_2023_34770_MOESM1_ESM.zip › Supplementary/Supplementary-Figures-1-5.pdf]

Supplementary Figure 1

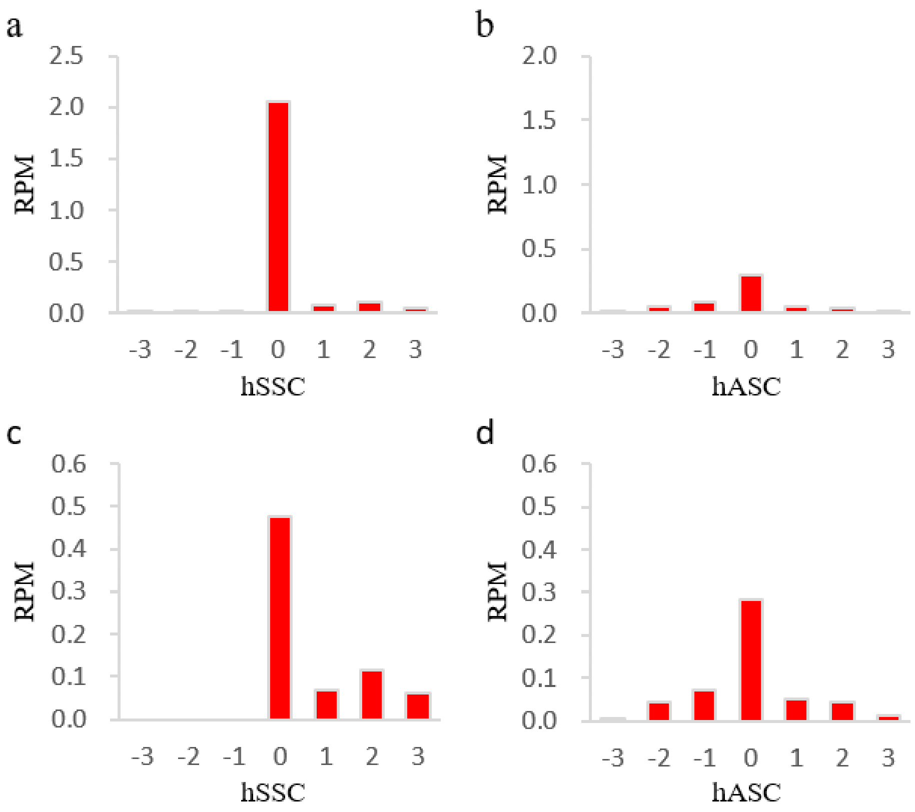

Supplementary Figure 1. Ribosome occupancy for the human-specific start codons and related positions in the four Yoruba individuals from dataset GSE61742. The mean ribosome occupancy was calculated across a) 19 human-specific start codons (hSSC) and b) the corresponding downstream human ancestral start codons. The mean ribosome occupancy was calculated across a) 19 human-specific start codons (hSSC) and b) the corresponding downstream human ancestral start codons. The mean ribosome occupancy was calculated across c) 16 non-CUG originated human-specific start codons (hSSC) and d) the corresponding downstream human ancestral start codons (hASC).

Supplementary Figure 2

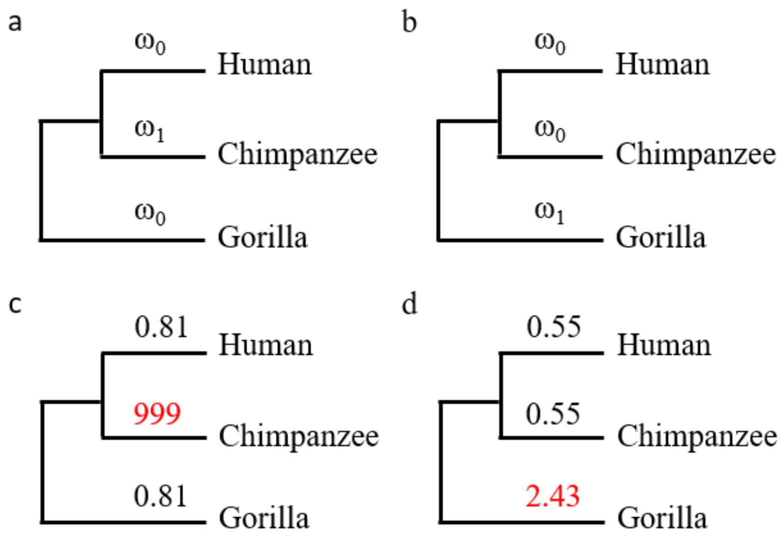

Supplementary Figure 2. Different branch models were compared to address whether the chimpanzee or gorilla branch had a lower ω value than the other branches. a) The third model assumed the chimpanzee branch had an independent ω value (ω_1) and the other branches shared the same ω value (ω_0). b) the forth model assumes the gorilla branch had an independent ω value (ω_1) and the other branches shared the same ω value (ω_0). The values of ω were estimated for c) the third model and d) the fourth model using PAML.

Supplementary Figure 3

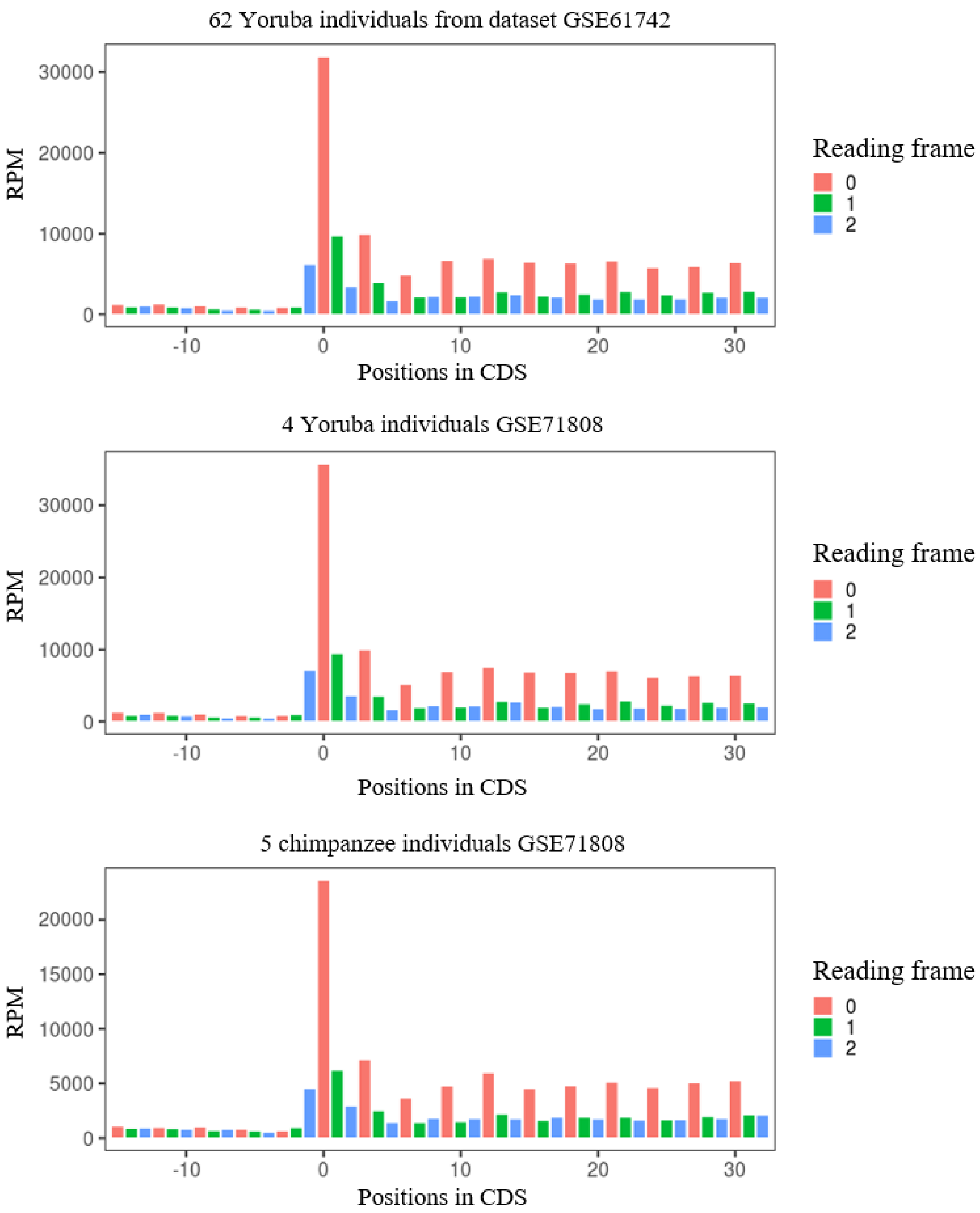

Supplementary Figure 3. Ribosome occupancy around start codons. The accumulated ribosome occupancy across all genes was calculated for a) 62 Yoruba individuals from dataset GSE61742, b) 4 Yoruba individuals from dataset GSE71808 and c) 5 chimpanzee individuals from dataset GSE71808. The X-axis represents the position in the coding sequence, and ‘0’ represents the first nucleotide of the start codon.

Supplementary Figure 4

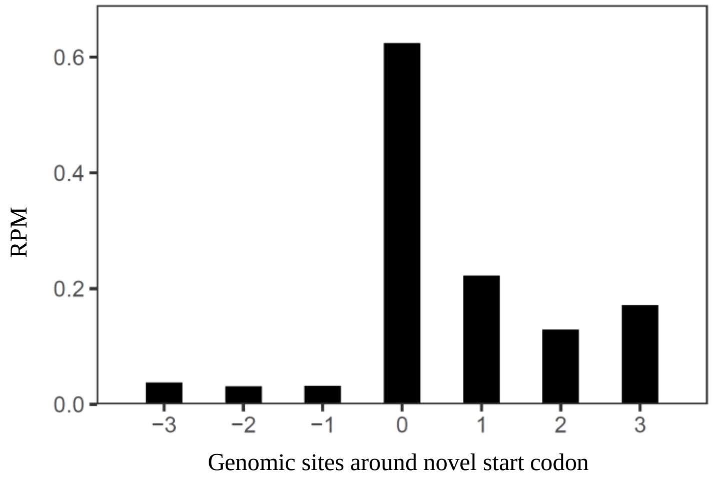

Supplementary Figure 4. Ribosome occupancy around start codons of all coding transcripts in the human genome.

Supplementary Figure 5

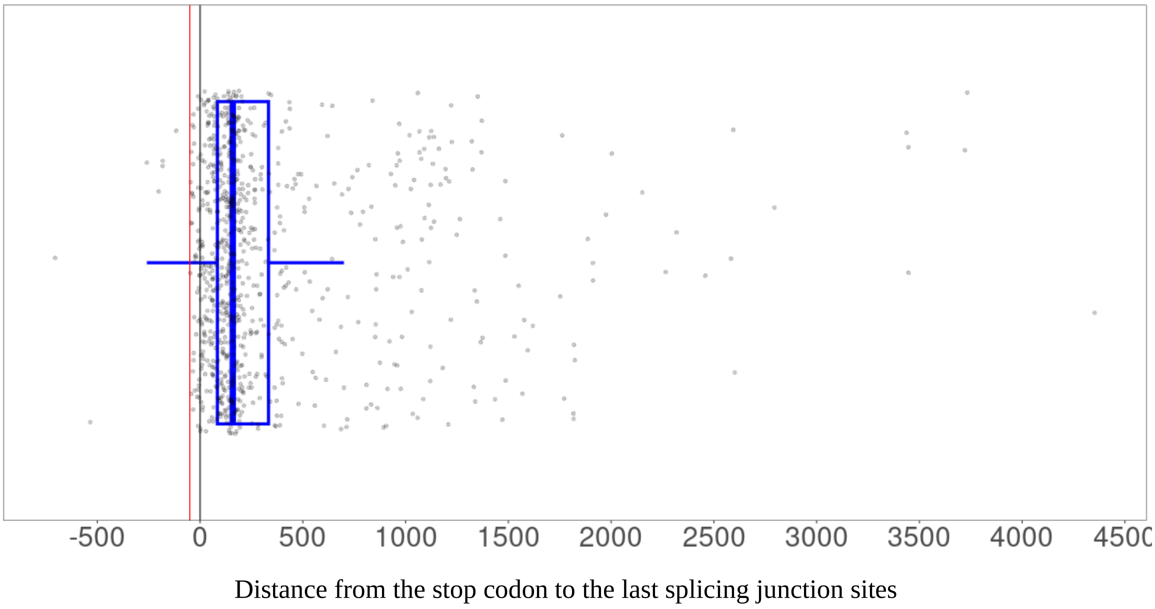

Supplementary Figure 5. Boxplot of the distance from stop codon to the last splicing junction site for all transcripts with start-gain SNVs in human genome. Blue line represents the position of the last splicing junction site in the transcript. Read line represents 50nt upstream of the last splicing junction site.
