# Supplementary material for: Novel start codons introduce novel coding sequences in the human genomes
Source: Sci Rep. 2023 May 19;13:8141. doi: 10.1038/s41598-023-34770-z (PMC10198996; doi:10.1038/s41598-023-34770-z)
Supplement: Supplementary file 1 — Supplementary Information 1. [file 41598_2023_34770_MOESM1_ESM.zip › Supplementary/Supplementary-Table-3.docx]

Supplementary Table 3. Statistics of the number of start-gain SNVs in individuals from different populations.

| Population | # of novel start codons  in each individual | | | # of novel start codons (AF>5%)  in each individual | | |
| --- | --- | --- | --- | --- | --- | --- |
|  | Min | Median | Max | Min | Median | Max |
| AFR (African) | 13 | 26 | 39 | 7 | 15 | 22 |
| AMR (Ad Mixed American) | 11 | 21 | 32 | 8 | 14 | 20 |
| EAS (East Asian) | 13 | 22 | 34 | 9 | 15 | 20 |
| EUR (European) | 12 | 21 | 32 | 7 | 14 | 22 |
| SAS (South Asian) | 12 | 23 | 33 | 7 | 15 | 21 |

* Populations were based on 1000 Genomes Project. ‘AF’ means ‘Allele Frequency’.
