# Supplementary figures and images for: Novel start codons introduce novel coding sequences in the human genomes
Source: Sci Rep. 2023 May 19;13:8141. doi: 10.1038/s41598-023-34770-z (PMC10198996; doi:10.1038/s41598-023-34770-z)

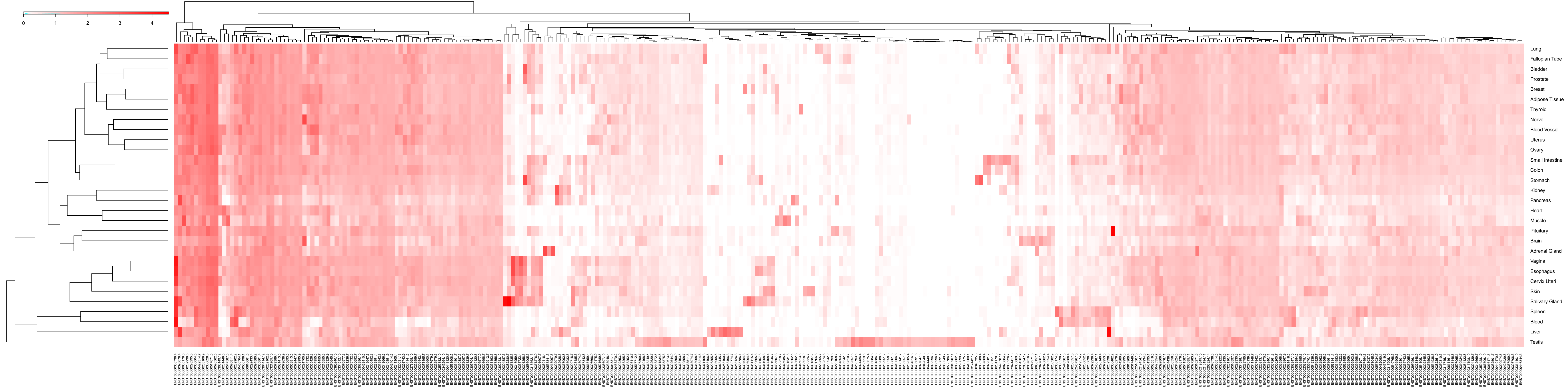

Supplement: Supplementary file 1 — Supplementary Information 1. [file 41598_2023_34770_MOESM1_ESM.zip › Supplementary/Supplementary-Figure-6..pdf]
